# Macrophages and Natural Killers Degrade α-Synuclein Aggregates

**DOI:** 10.1021/acs.molpharmaceut.4c00160

**Published:** 2024-04-18

**Authors:** Mikhail Matveyenka, Kiryl Zhaliazka, Dmitry Kurouski

**Affiliations:** † Department of Biochemistry and Biophysics, Texas A&M University, College Station, Texas 77843, United States; ‡ Department of Biomedical Engineering, Texas A&M University, College Station, Texas 77843, United States

**Keywords:** macrophages, natural killers, α-synuclein, cholesterol, phospholipids, amyloids

## Abstract

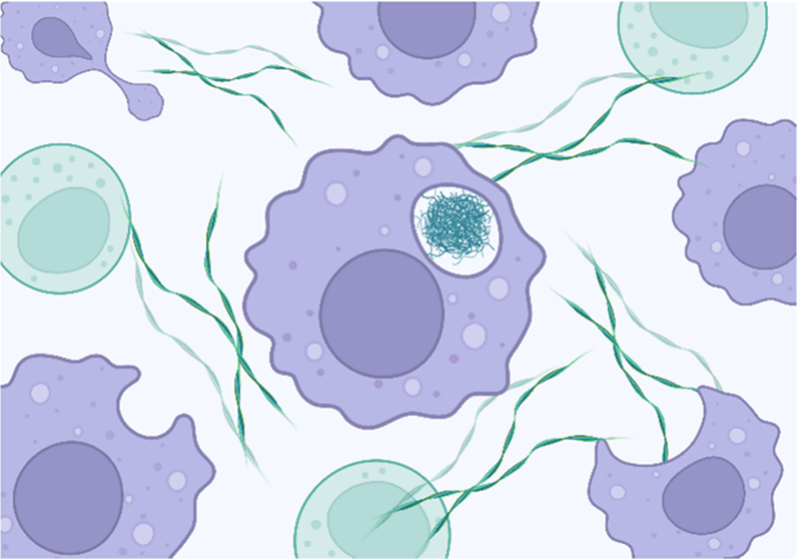

Amyloid oligomers and fibrils are protein aggregates that exert a high cell toxicity. Efficient degradation of these protein aggregates can minimize the spread and progression of neurodegeneration. In this study, we investigate the properties of natural killer (NK) cells and macrophages in the degradation of α-synuclein (α-Syn) aggregates grown in a lipid-free environment and in the presence of phosphatidylserine and cholesterol (PS/Cho), which are lipids that are directly associated with the onset and progression of Parkinson’s disease. We found that both types of α-Syn aggregates were endocytosed by neurons, which caused strong damage to cell endosomes. Our results also indicated that PS/Cho vesicles drastically increased the toxicity of α-Syn fibrils formed in their presence compared to the toxicity of α-Syn aggregates grown in a lipid-free environment. Both NK cells and macrophages were able to degrade α-Syn and α-Syn/Cho monomers, oligomers, and fibrils. Quantitative analysis of protein degradation showed that macrophages demonstrated substantially more efficient internalization and degradation of amyloid aggregates in comparison to NK cells. We also found that amyloid aggregates induced the proliferation of macrophages and NK cells and significantly changed the expression of their cytokines and chemokines.

## Introduction

Parkinson’s disease (PD) is the fastest-growing neurodegenerative disease and is projected to affect 12 million people worldwide by 2040.^1^ There are 60 000 cases of PD diagnosed annually in the US, with estimated costs that are upward of 30 billion, making effective neuroprotective treatments an urgent and unmet need.^2^ Unfortunately, current treatments focus on mitigating the motor dysfunction associated with PD and are not neuroprotective.^3,4^ PD is caused by the sequential loss of dopaminergic (DA) neurons in the substantia nigra pars compacta (SNc). Although the exact cause of the progressive neurodegeneration of DA neurons remains unclear, there is a growing body of evidence suggesting that the abnormal aggregation of α-synuclein (α-Syn) is the underlying molecular cause of PD.

α-Syn is a small 14 kDa protein that regulates neurotransmitter release by synaptic vesicles.^5−8^ Under pathological conditions, it aggregates and forms soluble oligomers with a variety of structures in vitro and in vivo.^9−15^ Some of these oligomers can propagate into fibrils that are long, unbranched β-sheet-rich assemblies.^16,17^ Microscopic analysis of α-Syn deposits in the midbrain, hypothalamus, and thalamus, which are known as Lewy bodies, revealed the presence of fragments of lipid membranes. These findings suggested that lipid membranes could play an important role in α-Syn aggregation. Galvagnion and colleagues found that lipids could accelerate or decelerate the rate of α-Syn aggregation.^18−20^ NMR and fluorescence methods revealed that the charged headgroups of lipids interacted with lysine and glutamic acid residues on the N-terminus (aa 1–60) of a-Syn.^21^ In parallel, the fatty acids of lipids developed hydrophobic interactions with the central part (aa 61–95) of α-Syn, which is also known as the NAC domain.^22,23^ These findings showed that the secondary structure of α-Syn/lipid aggregates directly depends on the chemical structure of the lipid.^24^ Our group showed that lipids altered both the rates of α-Syn aggregation and the secondary structure and toxicity of protein oligomers formed in the early^25^ and late^26^ stages of protein aggregation. Similar findings were reported by Matveyenka and colleagues for other amyloidogenic proteins, such as insulin and lysozyme.^27−30^ Specifically, it has been shown that phosphatidylcholine (PC) and phosphatidylserine (PS) could drastically alter the toxicity of insulin and lysozyme aggregates grown in the presence of these lipids.^31^ Furthermore, Jakubec and colleagues demonstrated that zwitterionic lipids, such as PC, strongly inhibited a-Syn aggregation, whereas cholesterol accelerated it.^24^ A growing body of evidence shows that cholesterol can modulate α-Syn aggregation, facilitating the interactions of α-Syn oligomers with plasma membranes, which causes membrane disruption and ultimately cell death.^32^ These interactions can be modulated by phospholipids such as PS.^32^ Although primarily localized at the inner part of plasma membranes under physiological conditions,^33−35^ negatively charged PS appears on the exterior membrane surface during cell dysfunction.^36^ In such cases, PS is recognized by phagocytes that degrade apoptotic and necrotic cells.^37^ Recently reported results from our group showed that the length and saturation of fatty acids (FAs) in PS altered the aggregation rate of α-Syn, as well as changed the toxicity of α-Syn oligomers and fibrils.^38^ Similar results were reported by Ali and co-workers for transthyretin (TTR).^39^ It was also shown that an increase in the concentration of cholesterol relative to PC in large unilamellar vesicles (LUVs) caused an increase in the aggregation rate of TTR.^40^ Furthermore, TTR fibrils formed in the presence of PC:cholesterol LUVs had substantially lower cytotoxicity compared to TTR fibrils formed in the lipid-free environment.^40^

Macrophages and natural killer (NK) cells play critical roles in the body’s early defense against numerous pathogens, including bacteria and viruses.^41−43^ They initiate and coordinate the immune response through cytokine and chemokine secretion.^44−47^ It was recently found that NK cells could efficiently internalize and clear α-Syn aggregates without aberrant activation and systemic depletion.^48^ Although it was suggested that NK cells use the endosomal/lysosomal pathway to degrade aggregates, the molecular mechanisms of this degradation remain unclear.^48^ A growing body of evidence suggests that macrophages can be used to clear amyloid aggregates.^49,50^ For instance, Richey and colleagues showed that macrophages could phagocytose immunoglobulin light chain fibrils.^49^ Gaiser and colleagues found that serum amyloid A1 (SAA1) aggregates polarized macrophages to the M1 state.^50^ This conclusion was made based on the observed secretion of the M1 cytokines TNF-α, IL-6, and MCP-1. These M1-polarized macrophages exhibited enhanced fibrillogenic activity toward SAA1 aggregates.^50^ In this study, we investigated the efficiency of NK cells and macrophages in degrading α-Syn oligomers and fibrils grown in a lipid-free environment and in the presence of phosphatidylserine and cholesterol (PS/Cho), which are lipids that are directly associated with the onset and progression of Parkinson’s disease. We also examined changes in the proliferation and cytokine profiles of the NK cells and macrophages.

## Experimental Section

### Materials

Human recombinant α-Syn was purchased from AnaSpec, CA, USA, and cholesterol (Cho) and 1,2-dimyristoyl-*sn*-glycero-3-phospho-l-serine (PS) were purchased from Avanti (Alabaster, Alabama).

### Liposome Preparation

PS and Cho at a 60:40 mol ratio were mixed in chloroform. Once all solvent was dried, the lipid mixture was dissolved in phosphate-buffered saline (PBS) pH 7.4. Next, the lipid solution was heated in a water bath to ∼50 °C for 30 min and then immersed in liquid nitrogen for 3–5 min. This procedure was repeated 10 times. Finally, the lipid solution was processed using an extruder equipped with a 100 nm membrane (Avanti, Alabaster, Alabama). Dynamic light scattering was used to ensure that the size of PS/Cho LUVs was within 100 ± 10 nm.

### Protein Aggregation

α-Syn was dissolved in PBS to reach the final protein concentration of 40 μM. In parallel, α-Syn and PS/Cho LUVs at a 1:1 molar ratio were dissolved in PBS. Next, samples were placed in a 96-well plate that was kept in the plate reader (Tecan, Männedorf, Switzerland) at 37 °C for 24 h under 510 rpm orbital agitation. Data were collected every 10 min.

### Kinetic Measurements

Rates of α-Syn and α-Syn/PS/Cho aggregation were measured using the thioflavin T (ThT) fluorescence assay. For this, samples were mixed with 2 mM ThT solution and placed into a 96-well plate that was kept in the plate reader (Tecan, Männedorf, Switzerland) at 37 °C for 24 h under 510 rpm agitation. Fluorescence measurements were taken every 10 min (excitation 450 nm; emission 488 nm).

### Atomic Force Microscopy (AFM) Imaging

Microscopic analysis of protein aggregates was performed on an AIST-NT-HORIBA system (Edison, NJ) using silicon AFM probes (force constant 2.7 N/m; resonance frequency 50–80 kHz) purchased from Appnano (Mountain View, California). Preprocessing of the collected AFM images was made using AIST-NT software (Edison, New Jersey).

### Attenuated Total Reflectance Fourier-Transform Infrared (ATR-FTIR) Spectroscopy

An aliquot of the protein sample was placed onto the ATR crystal and dried at room temperature. Spectra were measured using a Spectrum 100 FTIR spectrometer (PerkinElmer, Waltham, Massachusetts). Three spectra were collected from each sample and averaged using Thermo Grams Suite software (Thermo Fisher Scientific, Waltham, Massachusetts).

### Cell Culturing

Mice midbrain N27 cells were purchased from ATCC and grown in RPMI 1640 Medium (Thermo Fisher Scientific, Waltham, Massachusetts) with 10% fetal bovine serum (FBS) (Invitrogen, Waltham, Massachusetts) in a 96-well plate (5000 cells per well) at 37 °C under 5% CO_2_. After 2 h, the cells were found to fully adhere to the wells, reaching ∼70% confluency.

Mice macrophages were purchased from ATCC and grown in DMEM with 10% fetal bovine serum (FBS) (Invitrogen, Waltham, Massachusetts) in a 96-well plate (5000 cells per well) at 37 °C under 5% CO_2_. Mice NK cells were purchased from ATCC and MyeloCult with 10% fetal bovine serum (FBS) (Invitrogen, Waltham, Massachusetts) and IL-2 (Sigma-Aldrich, St. Louis, MO) in a 96-well plate (5000 cells per well) at 37 °C under 5% CO_2_.

### Cell Toxicity Assay

After 24 h of incubation with the sample of the protein aggregates, the cells were stained using Annexin V cell viability assay and analyzed on an LSRII BD flow cytometer (BD, San Jose, California). Cell viability was determined by using LSRII software.

### Membrane Leakage Assay

Plasmids that code charged multivesicular body protein 1b (Chmp1b, cell membrane repair), Galectin-3 (Gal3, autophagy), and transcription factor EB (TFEB, lysosome biogenesis) were delivered to HEK 293T cell using GeneX Plus reagent (ACS-4004, ATCC, Manassas, Virginia). The cells were grown in Dulbecco’s Modified Eagle Medium (DMEM) cell medium that contained 10% FBS. After 24 h, HEK 293T cells were found to reach ∼80% confluency. Transfection was made in DMEM without FBS. Next, cell media was replaced on DMEM with 10% FBS and incubated for 24 h. Protein samples were added to the cells and incubated for 24 h. Fluorescence cell imaging was performed in an EVOS M5000 microscope (Thermo Fisher Scientific, Waltham, Massachusetts). Chmp1b and TFEB plasmids contain green fluorescence protein, and Gal3 contains red fluorescence protein. Microscopic images were used to count the number of punctata in cells treated with protein aggregates grown in the presence and absence of PS/Cho.

### Proliferation Assay

After 24 h of incubation with the sample of the protein aggregates, the cells were stained using CellTiter 96 nonradioactive cell proliferation assay (MTT) kit (Promega, Madison, Wisconsin). Cells were incubated with the dye for 4 h at 37 °C under 5% CO_2_. Next, solubilization solution (Promega, Madison, Wisconsin) was added and incubated for 1 h at 37 °C under 5% CO_2_. Absorption measurements were made in a plate reader (Tecan, Männedorf, Switzerland) at 570 nm. Every well was measured 25 times in different locations.

### Expression of Cytokines

After incubation, the cells were collected for the isolation of ribonucleic acid (RNA) by extraction using Trizol (Invitrogen). To determine the presence and amount of RNA, we used agarose gel electrophoresis and the agarose gel documentation system as well as NanoDrop. Next, we synthesized coding deoxyribonucleic acid (DNA) using reverse transcriptase, a set of SuperScript III (Invitrogen). A real-time quantitative polymerase chain reaction (q-PCR) was performed with primers corresponding to selected genes. SYBR Master Mix (Thermo Fisher Scientific) was used for q-PCR of the mixture. q-PCR was performed with primers previously reported by Matveyenka and co-workers in the CFX96 Real-Time System (Bio-Rad) device.^51^

Each sample was performed in a triplet. Then we plotted the average CT values from each sample compared to the absolute amount of the control gene, the so-called housekeeping genes; in our case, it was *GAPDH* and *β-Actin*, to create a standard curve. This comparison of experimental CT data with the control gene gives the value of the number of genes of interest to us that are present in the cells. For data analysis, the values of samples were selected that exhibited a growth of up to 35 cycles, control genes—up to 25 cycles. In each triplet, the variation between repetitions was up to 0.5 cycles. When the data were analyzed, we used the −2^ΔCT^ method.

### Enzyme-Linked Immunoassay (ELISA)

ELISA assay was performed by using an α-Syn ELISA Kit (Invitrogen, cat.N. KHB0061). Cells were concentrated by centrifugation and lysed using Cell Extraction Buffer (10 mM Tris (pH 7.4), 100 mM NaCl, 1 mM EGTA, 1 mM NaF, 20 mM Na_2_P_2_O_7_, 2 mM Na_3_VO_4_, 1% Triton X-100, 10% glycerol, 0.1% SDS, and 0.5% deoxycholate) for 30 min, on ice, with mixing on vortex at 10 min intervals. Then, lysate was centrifuged at 13 000 rpm for 10 min at 4 °C, and the supernatant was saved.

After standards preparation, 50 μL of standards, controls, and samples were added to wells, except for chromogen blanks. Then, 50 μL of Hu-α-Synuclein Detection Antibody solution was added to each well except for the chromogen blanks. After incubation for 3 h at room temperature, the solution was aspirated from wells, and the wells were washed 4 times with wash buffer. Next, 100 μL of anti-rabbit IgC HRP was added into each well except for the chromogen blanks. The plate was incubated at room temperature for 30 min. After that solution was aspirated, and the wells were washed 4 times with wash buffer. Then, 100 μL of stabilized chromogen was added to each well. After incubation for 30 min in the dark, 100 μL of stop solution was added to each well. Absorbance was measured at 450 nm.

## Results and Discussion

### Structural Characterization of α-Syn Aggregates Grown in the Presence of Lipids and in a Lipid-Free Environment

**Figure 1:**
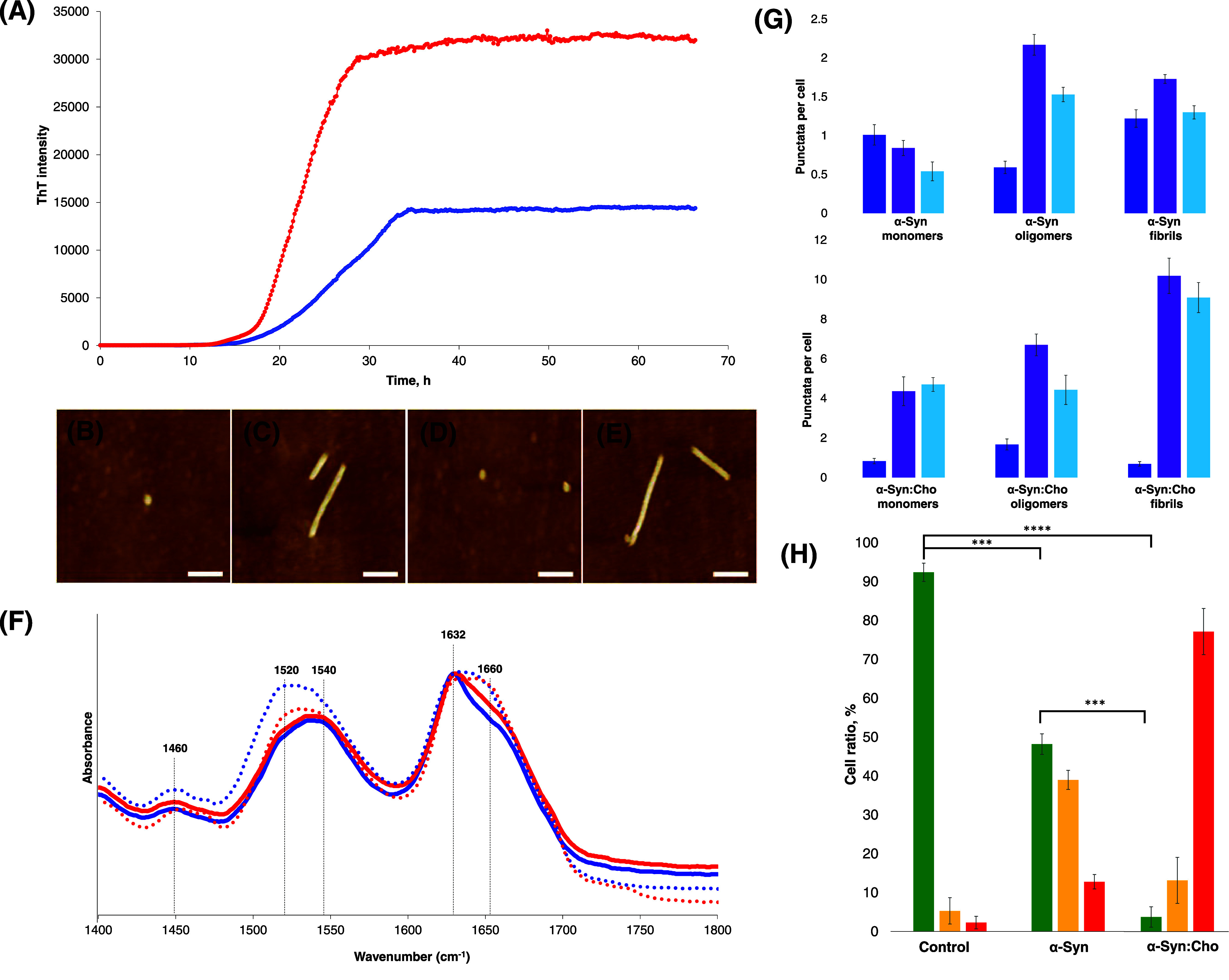
Lipids alter the rate of α-Syn aggregation, yielding more toxic oligomers and fibrils. Averaged ThT aggregation kinetics (A) of α-Syn aggregation in the lipid-free environment (blue) and in the presence of PS/Cho (red). All measurements were made in triplicates. AFM images of α-Syn oligomers (B) and fibrils (C), as well as α-Syn/PS/Cho oligomers (D) and fibrils (E). FTIR spectra (F) of α-Syn oligomers (dashed blue) and fibrils (solid blue) and α-Syn/PS/Cho oligomers (dashed red) and fibrils (solid red). Histograms (G) of fluorescent puncta per cell of Chmp1 (blue) and Gal3 (purple), as well as the sum of pixels from fluorescent TFEB (light blue) puncta. For each of the presented results, at least 15 individual images of N21 rat dopaminergic cells were analyzed. Histograms (H) of N27 rat dopaminergic cell viability according to Annexin V cell viability assay. Live cells are shown in green, apoptotic cells are shown in yellow, and dead cells are shown in red.

α-Syn aggregation at pH 7.4 is characterized by a lag phase (*t*_lag_ = 14.5 ± 0.8 h) that is followed by a rapid increase in ThT fluorescence, which indicates the formation of fibrils (Figure 1A). In the presence of an equimolar concentration of PS/Cho, the lag phase (*t*_lag_ = 12.1 ± 0.1 h) was significantly shorter, which indicated that these lipids accelerated α-Syn aggregation. We also observed a much greater intensity of protein aggregates that formed in the presence of PS/Cho compared to that in the lipid-free environment. This observation demonstrates that more ThT-active protein aggregates are formed in the presence of PS/Cho. Alternatively, the presence of lipids could increase the binding affinity of ThT to the surface of protein aggregates.

Morphological and structural analyses of α-Syn and α-Syn/PS/Cho aggregates formed at ∼20 h (lag phase) and ∼50 h (plateau) revealed the presence of spherical oligomers and fibrils, respectively (Figure 1B–E). We found that oligomers exhibited heights of ca. 6–8 nm, whereas α-Syn and α-Syn/PS/Cho fibrils were 10–12 nm in height. In the IR spectra of both α-Syn and α-Syn/PS/Cho oligomers, we observed equally intense vibrational bands centered at 1630 and 1660 cm^–1^, which could be assigned to parallel β-sheet and unordered protein secondary structures (Figure 1F). Based on these results, we could conclude that α-Syn and α-Syn/PS/Cho oligomers contained parallel β-sheet and unordered protein secondary structures. IR spectra collected from both α-Syn and α-Syn/PS/Cho fibrils exhibited an intense peak at 1630 cm^–1^ with a shoulder at 1660 cm^–1^. These findings demonstrated that α-Syn and α-Syn/PS/Cho fibrils formed at 50 h of protein aggregation had predominantly parallel β-sheet secondary structures. These results also showed that not only the morphology (Figure 1B–E) but also the secondary structures of α-Syn and α-Syn/PS/Cho fibrils were very similar.

Amyloid aggregates can accumulate inside endosomes, causing damage to endosomal membranes.^52^ As a result, protein aggregates leak into the cytosol, where they induce ER and mitochondrial dysfunction and enhance ROS production.^53,54^ We performed a set of biochemical assays to determine the extent to which α-Syn and α-Syn/PS/Cho monomers, oligomers, and fibrils damage endosomal membranes. Endosomal membrane damage can be quantified using the markers Chmp1-EGFP, Gal3-EGFP, and TFEB-EGFP. Chmp1 proteins bind to the membranes of damaged endosomes that exhibit Ca^2+^ leakage into the cytosol, engaging the ESCRT-III complex in membrane repair processes.^55−57^ Endosomal damage can also expose luminal β-galactosides to the cytosol. Cytosolic Gal3 binds to exposed β-galactosides, thereby initiating autophagy.^55,58^ In the absence of endosomal damage, diffuse and cytosolic distribution of Chmp1/Gal3 is observed, whereas amyloid-induced damage to endosomal membranes results in the appearance of Chmp1/Gal3-EGFP puncta, which can be quantified using fluorescence microscopy. Such damage initiates endosomal repair, the clearance of damaged endosomes by autophagy, and de novo biogenesis of organelles.^55^ TFEB is a transcription factor that regulates lysosomal biogenesis and autophagy.^59−61^ Activation of TFEB is linked to endosomal Ca^2+^ efflux, activation of the phosphatase calcineurin, the dephosphorylation of TFEB, and the subsequent translocation of the transcription factor to the nucleus.^55^ In turn, nuclear TFEB activates a transcriptional program that induces de novo biogenesis of endosomal organelles. Therefore, cells transfected with TFEB-EGFP were used to monitor the cytoplasm-to-nucleus translocation of the transcription factor in response to exposure to α-Syn and α-Syn/PS/Cho monomers, oligomers, and fibrils.

It was found that α-Syn and α-Syn/PS/Cho monomers, oligomers, and fibrils caused severe damage to endosomal membranes (Figure 1G). The magnitude of endosomal damage directly depended on the protein aggregation state and the presence of PS/Cho during α-Syn aggregation. Specifically, we found that α-Syn oligomers formed in the lipid-free environment primarily induced endosomal autophagy (Gal3) and de novo endosome biogenesis (TFEB), whereas α-Syn fibrils nearly equally engaged Ca^2+^ leakage, endosomal repair (Chmp1b) and de novo endosome biogenesis (TFEB). Similar signs of endosomal damage were observed in response to the α-Syn monomers. These results suggested that α-Syn monomers accumulated and aggregated in the endosomes. Furthermore, the magnitude of endosomal damage caused by α-Syn/PS/Cho monomers, oligomers, and fibrils was 4–10 times stronger than the damage caused by the corresponding α-Syn species. Our findings showed that α-Syn/PS/Cho monomers, oligomers, and fibrils triggered endosomal autophagy (Gal3) and de novo biogenesis (TFEB) with only small engagement of Ca^2+^ leakage (Chmp1b). These results demonstrated that the presence of PS/Cho drastically changed the physiological response of cells to these protein species. These results also suggested that α-Syn/PS/Cho exerted higher cell toxicity in comparison to α-Syn aggregates grown in the absence of lipids.

We used N27 mouse rat neuronal cells to determine the toxicity of α-Syn and α-Syn/PS/Cho aggregates. We found that α-Syn fibrils lowered cell viability from ∼95% (control) to ∼50% (Figure 1H). Furthermore, α-Syn/PS/Cho aggregates were found to be substantially more toxic to N27 cells than α-Syn fibrils. Specifically, we found that >90% of cells were apoptotic or dead after exposure to α-Syn/PS/Cho aggregates for 24 h. Thus, we can conclude that α-Syn/PS/Cho aggregates are much more toxic than α-Syn species formed in a lipid-free environment.

### Characterization of the Clearance Properties of Macrophages and NK Cells

**Figure 2:**
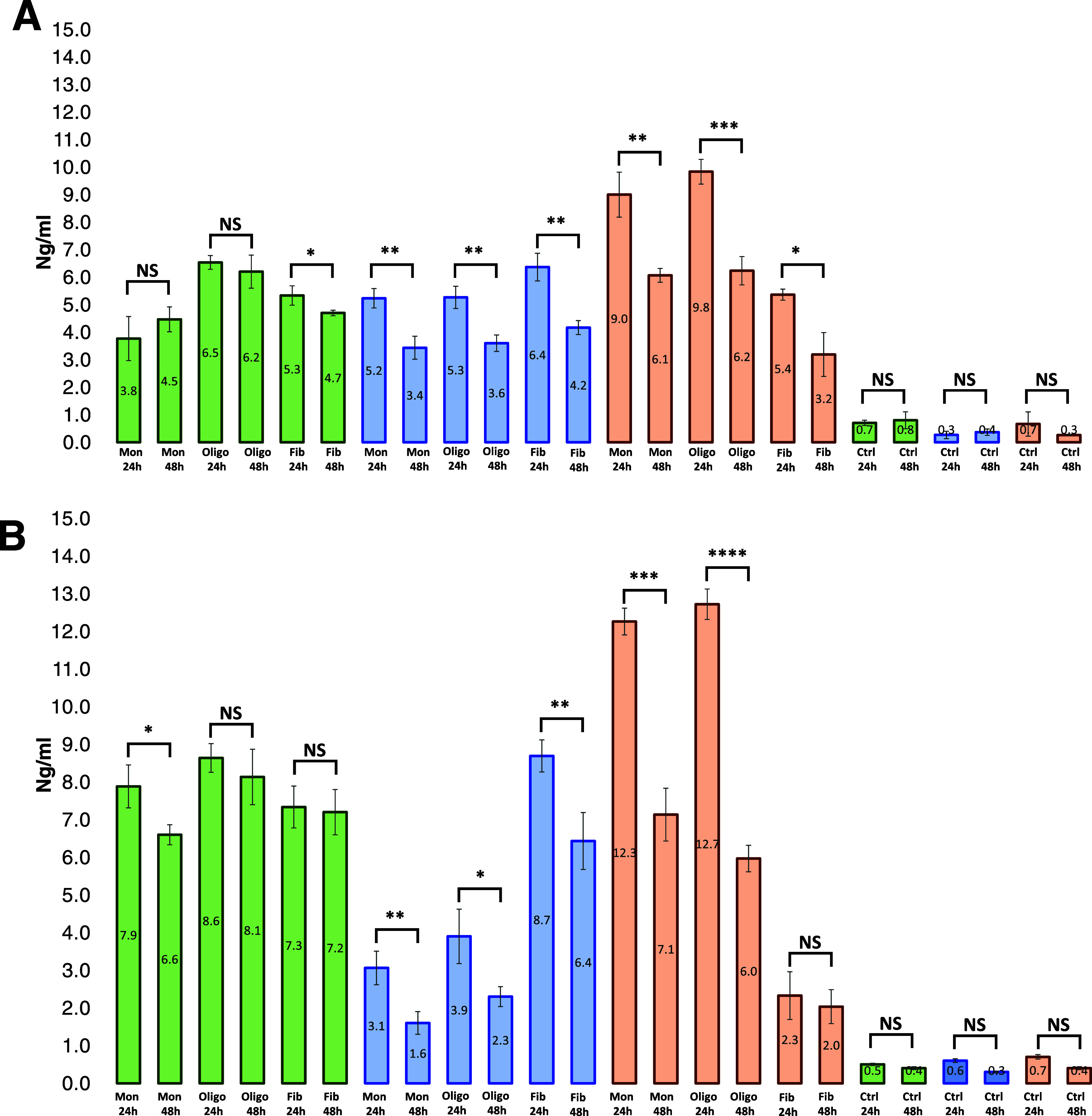
Macrophages and NK cells degrade amyloid aggregates. Histograms of ELISA results showing the concentration (ng/mL) of α-Syn (A) and α-Syn/PS/Cho (B) monomers (Mon), oligomers (Oligo), and fibrils (Fib) in N27 rat dopaminergic cells (green), NK (blue), and macrophages (orange) after 24 and 48 h.

Macrophages and NK cells, as well as the N27 mouse neuronal cell line discussed above (used as a control), were incubated with α-Syn and α-Syn/PS/Cho monomers, oligomers, and fibrils for 24 h. Next, protein monomers and aggregates were removed from the cell medium in three consecutive centrifugation steps. Half of the collected macrophages and NK and N27 cells were lysed to determine the concentration of the internalized protein, and the other half was cultured in amyloid-free medium for an additional 24 h. We used ELISA to quantify the amount of α-Syn and α-Syn/PS/Cho monomers, oligomers, and fibrils in the cells at 24 and 48 h (Figure 2).

We found that a substantial amount of protein monomers and aggregates accumulated in macrophages, NK cells, and N27 cells at 24 h. We also observed a drastic decrease in the concentration of α-Syn monomers, oligomers, and fibrils in macrophages after 48 h compared to the amount of protein aggregates in these cells at 24 h (Figure 2A). A similar decrease in the concentration of protein monomers and aggregates was observed in the NK cells. These results showed that both macrophages and NK cells were able to degrade α-Syn monomers, oligomers, and fibrils. Furthermore, an insignificant change in the concentration of α-Syn aggregates was observed in N27 cells after 24 and 48 h. These results showed that unlike macrophages and NK cells, N27 cells were not capable of degrading α-Syn monomers and protein aggregates.

We also investigated the relationship between the aggregation state of α-Syn/PS/Cho and their uptake by macrophages, NK cells, and N27 cells (Figure 2B). We found that N27 cells nearly equally endocytosed α-Syn/PS/Cho monomers, oligomers, and fibrils. However, NB cells demonstrated very strong endocytosis of fibrils compared to monomers and oligomers. Macrophages, in contrast, were able to take up very few fibrils, whereas the greatest endocytosis activity was observed in response to monomers and oligomers. We found a significant decrease in the concentration of α-Syn/PS/Cho monomers, oligomers, and fibrils in macrophages after 48 h (Figure 2B). Similar changes in the concentrations of these protein species were observed in NK cells. These results showed that both macrophages and NK cells were able to clear α-Syn/PS/Cho monomers, oligomers, and fibrils. Furthermore, nonsignificant changes in the concentrations of α-Syn/PS/Cho aggregates were observed in N27 cells at 48 h. These results showed that N27 cells could not degrade α-Syn/PS/Cho aggregates.

### Cell Proliferation and Amyloid-Induced Changes in the Cytokine and Chemokine Profiles of Cells

We investigated the responses of N27 cells, macrophages, and NK cells to α-Syn and α-Syn/PS/Cho aggregates. We measured cell proliferation rates and amyloid-induced changes in the cytokine and chemokine profiles of N27 cells, macrophages, and NK cells.

**Figure 3:**
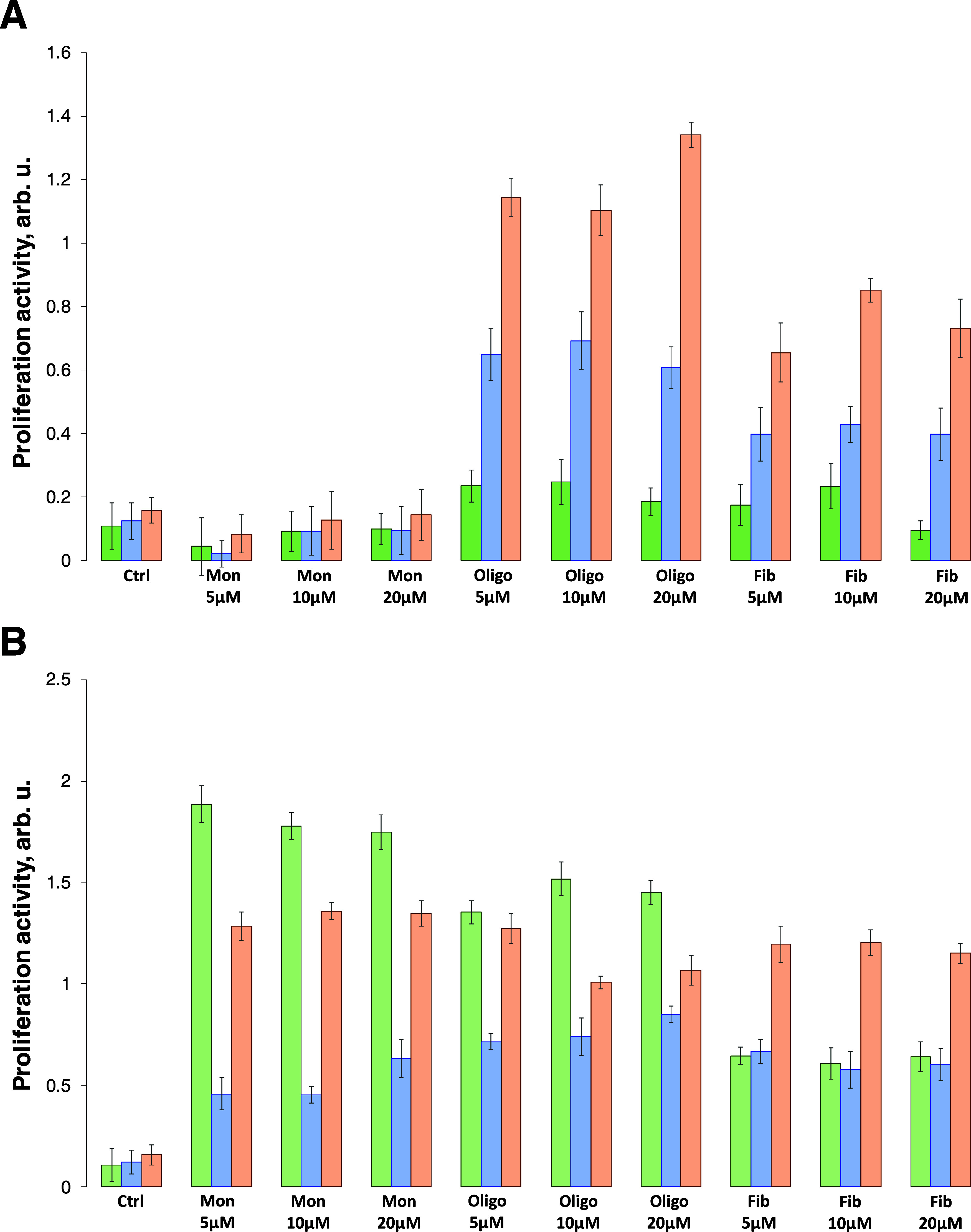
Amyloid aggregates grown in a lipid-free environment alter cell proliferation. Histograms of proliferation activity of N27 rat dopaminergic cells (green), NK (blue), and macrophages (orange) as a response to α-Syn monomers oligomers and fibrils at 24 h (A) and 48 h (B).

We found that exposing N27 cells, macrophages, and NK cells to α-Syn monomers caused no changes in cell proliferation at 24 h (Figure 3). These findings demonstrated that α-Syn monomers did not induce substantial changes in cell behavior. Furthermore, α-Syn oligomers and fibrils enabled strong proliferation in macrophages and NK cells. α-Syn fibrils induced significantly less proliferation in macrophages and NK cells than α-Syn oligomers. It should be noted that we observed no significant changes in the proliferation of N27 cells. Furthermore, analysis of cell proliferation at 48 h revealed a drastic increase in the proliferation of N27 cells, macrophages, and NK cells in response to α-Syn monomers, oligomers, and fibrils. These results suggest that α-Syn monomers accumulate in cells, which likely triggers their aggregation into oligomers and fibrils. Our findings also show that the presence of α-Syn aggregates in N27 cells, macrophages, and NK cells significantly changes their proliferation activity.

**Figure 4:**
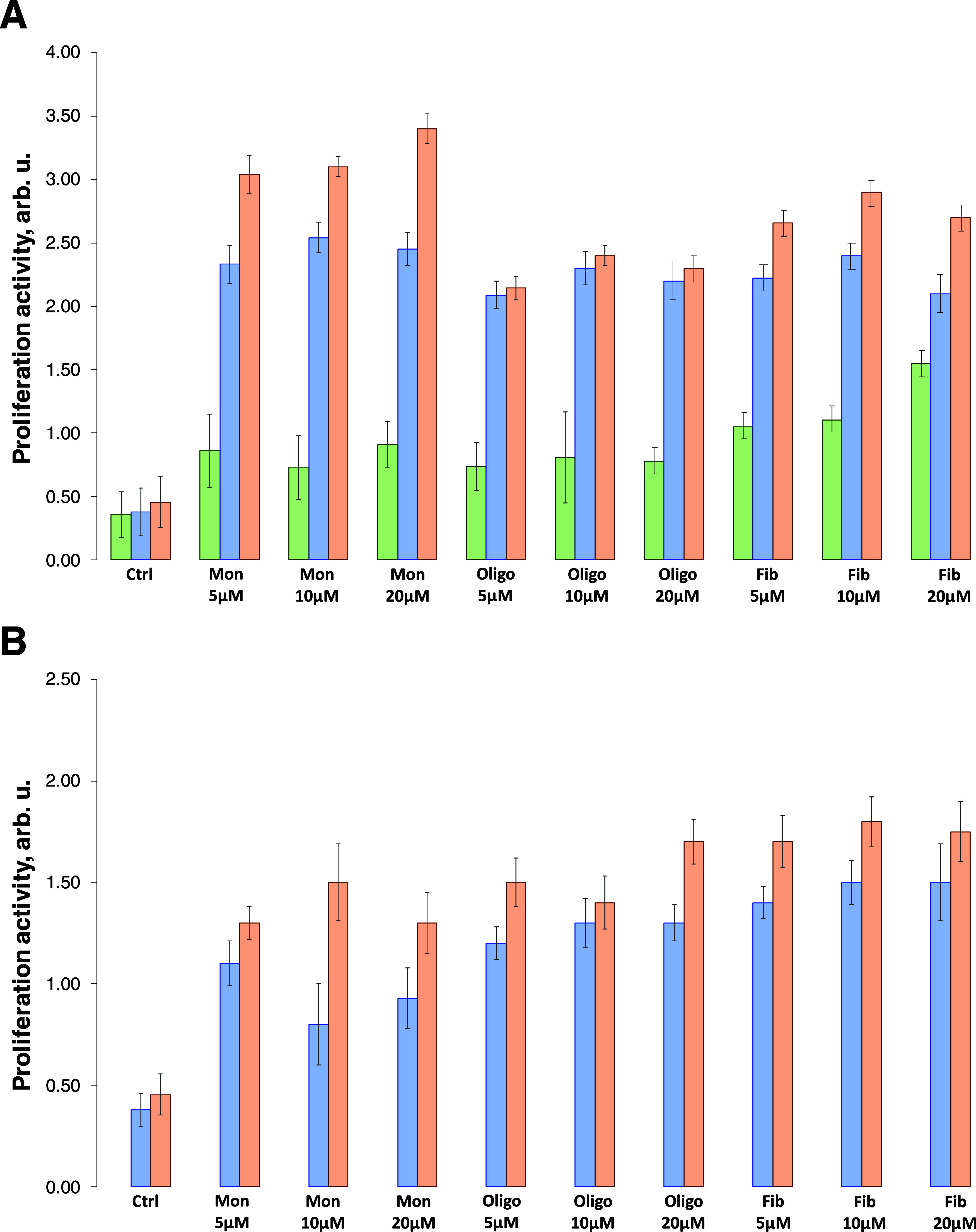
Amyloid aggregates grown in the presence of lipids alter cell proliferation. Histograms of proliferation activity of N27 rat dopaminergic cells (green), NK (blue), and macrophages (orange) as a response to α-Syn/PS/Cho monomers oligomers and fibrils at 24 h (A) and 48 h (B).

We observed significantly different responses in macrophages and NK cells to α-Syn/Cho monomers, oligomers, and fibrils (Figure 4). Specifically, we found that α-Syn/Cho monomers caused nearly 6-fold stronger activation of macrophages and 3-fold NK-cell proliferation than α-Syn monomers. We also found that exposing macrophages to α-Syn/Cho oligomers enabled strong cell proliferation that had a lower intensity than the monomer-induced proliferation of macrophages. Finally, fibrils induced similar levels of macrophage proliferation as monomers. Interestingly, NK cells exhibited similar enhancements in proliferation in response to α-Syn/Cho monomers, oligomers, and fibrils. After 48 h of exposure to α-Syn/Cho monomers, oligomers, and fibrils, we found that macrophages and NK cells had high levels of proliferation. However, we were not able to measure the levels of proliferation in N27 cells due to the strong toxicity of α-Syn/Cho aggregates to these cells (Figure S1). These results demonstrated that α-Syn/Cho induced significantly more proliferation in macrophages and NK cells than α-Syn monomers, oligomers, and fibrils. We infer that the change in cell proliferation activity is directly linked to the toxicity of α-Syn and α-Syn/Cho species.

**Figure 5:**
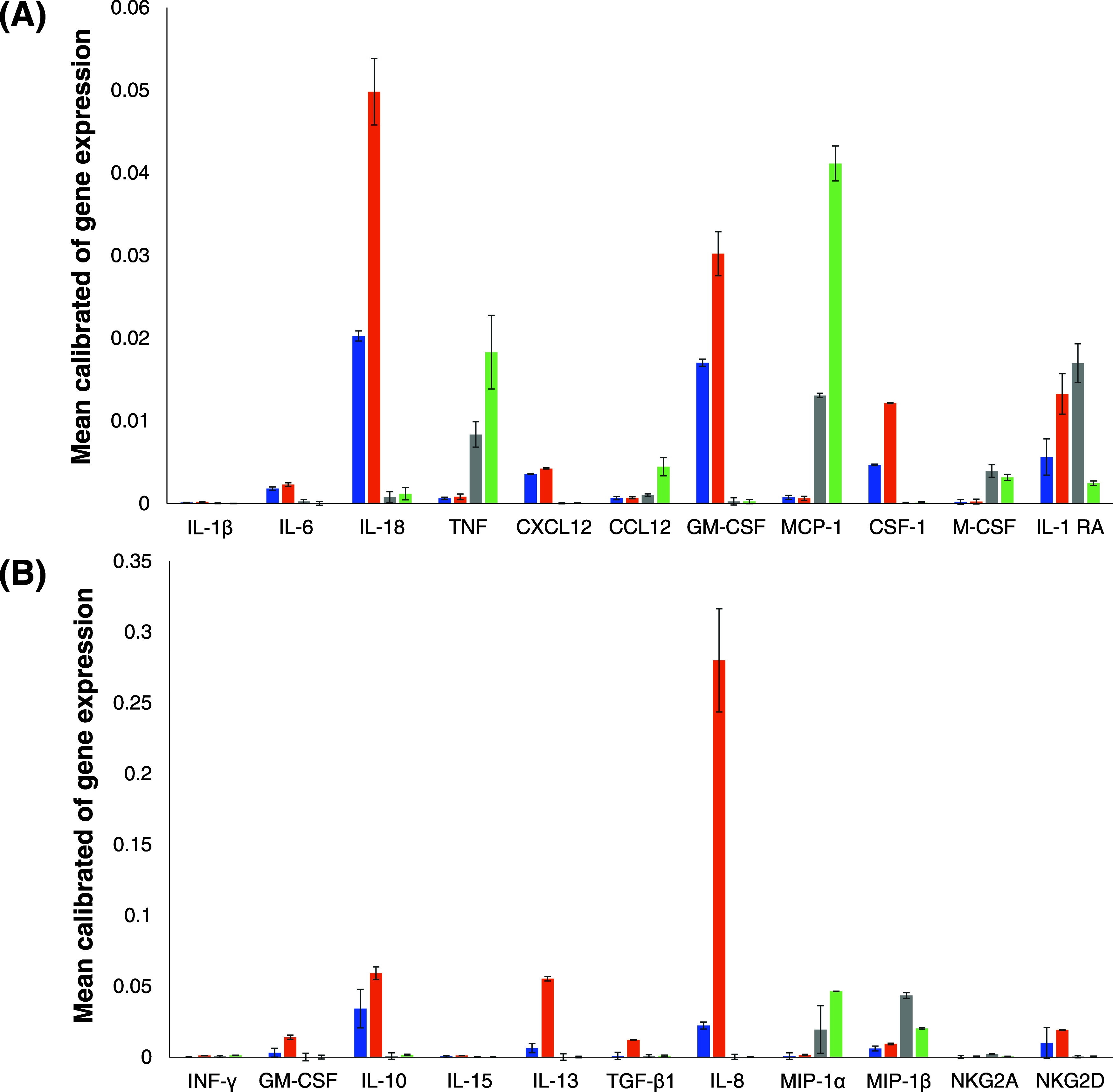
α-Syn monomers and aggregates alter the cytokine and chemokine profiles of macrophages and NK cells. Mean calibration of gene expression as the response of macrophages (A) and NK (B) cells to α-Syn monomers (blue), oligomers (orange), and fibrils (gray). Control levels of gene expression are shown in green.

In response to contact with pathogens and toxic species, macrophages release cytokines and chemokines to initiate the innate immune response (Figure 5).^62^ TNF, interleukin-1 β (IL-1β), interleukin 6 (IL-6), and interleukin 18 (IL-18) are key inflammatory cytokines in macrophages.^62−64^ The release of these cytokines initiates a potent defensive inflammatory response.^42,43^ IL-1Ra is an inhibitor of IL-1β that binds to the IL-1R1 receptor with high affinity, thereby competing with IL-1β.^65^

Monocyte chemoattractant protein-1 (MCP-1) is one of the key chemokines that regulates the migration and infiltration of macrophages.^66^ Macrophage colony-stimulating factor (GM-CSF) activates macrophage differentiation into inflammatory cells (M1).^67^ M-CSF, in contrast, promotes macrophage differentiation into anti-inflammatory phenotype (M2).^45−47^ Colony-stimulating factor (CSF)-1 is a hemopoietic growth factor for mononuclear phagocyte lineage cells that regulates macrophage differentiation, proliferation, and survival.^68,69^ It elicits its effect by binding with the CSF-1 receptor (CSF-1R), a high-affinity receptor tyrosine kinase encoded by the c-fms proto-oncogene.^70^ The chemokine CXCL12 (stromal cell-derived factor-1, SDF-1) is expressed under homeostatic conditions at sites where resident macrophages are present.^71^ CXCL12 plays an important role in monocyte extravasation.^71,72^ Therefore, it is typically produced under pathologic conditions involving ischemia and/or hypoxia. Ccl12 is a proinflammatory cytokine that is secreted by macrophages to impair fibronectin, and collagen deposition indirectly stimulates collagen degradation through the upregulation of matrix metalloproteinase-2 (Figure 5).^73^

We found that macrophages exerted drastically different cytokine and chemokine responses to α-Syn, oligomers, and monomers (Figure 5). Specifically, we observed a drastic increase in macrophage expression of inflammatory IL-6 and IL-18 in response to monomers and oligomers, whereas no activation of these cytokines was observed in response to α-Syn fibrils. We also observed significantly stronger macrophage activation of IL-18 expression in response to interactions with oligomers than with fibrils. Furthermore, α-Syn monomers and oligomers caused strong suppression of TNF expression; however, significantly higher expression levels of TNF were observed for α-Syn fibrils than the controls. Similar changes in the expression of MCP-1 were observed in response to fibrils compared to monomers and oligomers. We also observed no changes in the expression of IL-1β in macrophages exposed to α-Syn fibril oligomers or monomers.

We also found that α-Syn oligomers and monomers enhanced the expression of GM-CSF, which demonstrates that these protein species activate macrophage differentiation into inflammatory cells (M1) (Figure 5). However, this M1 polarization was not evident in response to the α-Syn fibrils. These results show that macrophages are specifically responsive to protein monomers and oligomers. These conclusions were further supported by the observed changes in the CSF-1 expression. We found that α-Syn oligomers and monomers caused drastic increases in the expression of this hemopoietic growth factor that regulates differentiation, proliferation, and survival in macrophages. However, no changes in the level of CSF-1 expression in macrophages were found in response to α-Syn fibrils. It is important to note that fibrils caused an increase in the expression levels of M-CSF that was not observed in response to α-Syn oligomers and monomers. These findings suggest that fibrils promote macrophage polarization toward the M2 phenotype.

NK cells initiate and coordinate the immune response through the secretion of several cytokines such as interferon (IFN)-γ, IL-10, IL-13, and IL-15 (Figure 5).^41^ The activity of NK cells can also be suppressed by TGF-β, an immunomodulatory cytokine with a prominent role in the adaptive immune response.^74^ TGF-β inhibits the production of (IFN)-γ, reducing the cytotoxic abilities of NK cells. IL-8, also termed CXCL8, is a chemokine that induces the migration of other immune cells, including T cells, NK cells, and monocytes.^41^ However, cytotoxic NK cells do not typically produce IL-8.^75^ Macrophage inflammatory protein-1 (MIP-1α) is another chemokine that regulates the migration of NK cells.^44^ It can be secreted by both macrophages and NK cells in a cytokine-dependent manner. Natural killer receptor group 2, member A (NKG2A) is a factor that indicates NK-cell exhaustion,^76^ and natural killer receptor group 2, member D (NKG2D) is a potent activating receptor that is expressed on all NK cells to detect cancer and viral cells.

We found that similar to macrophages, α-Syn monomers, oligomers, and fibrils caused drastically different cytokine and chemokine responses in NK cells (Figure 5). Specifically, we found that α-Syn oligomers enabled the strongest expression of IL-10, IL-13, and IL-15, as well as TGF-β. We also found no expression of (IFN)-γ in NK cells, which is consistent with the enhanced expression levels of TGF-β. We observed much weaker expression of IL-10, IL-13, and IL-15, as well as TGF-β, in NK cells in response to α-Syn monomers and the absence of any expression of these interleukins in response to α-Syn fibrils. Furthermore, α-Syn fibrils strongly activated MIP-1α, which promotes NK migration toward these protein aggregates. Finally, we observed no expression of NKG2A by NK cells in response to their interactions with α-Syn monomers, oligomers, and fibrils. These results demonstrate that the cytokine and chemokine profiles of macrophages and NK cells significantly change after interactions of these cells with α-Syn monomers, oligomers, and fibrils grown in the lipid-free environment, as well as in the presence of PS and Cho.

## Discussion

A growing body of evidence suggests that lipids can alter the rate of protein aggregation. For instance, Zhaliazka and co-workers found that zwitterionic lipids, such as phosphatidylcholine (PC) and phosphatidylethanolamine (PE) decelerated the rate of lysozyme aggregation, whereas negatively charged phosphatidylserine (PS), phosphatidylglycerol (PG), and cardiolipin (CL) accelerated fibril formation.^77^ Similar results were recently reported by Matveyenka and co-workers for insulin. It was also found that lipids uniquely altered secondary structure and toxicity of amyloid oligomers and fibrils.^51^ Specifically, insulin aggregation in the presence of PC yielded small oligomers that possessed a primarily unordered protein secondary structure. These aggregates exerted significantly lower cell toxicity compared to the insulin fibril formed in the lipid-free environment. Recently, Zhaliazka and co-workers demonstrated that Cho could drastically enhance the toxicity of amyloid β_1–42_ oligomers formed in its presence.^78^ Our results demonstrate that Cho also drastically enhanced the toxicity of α-Syn aggregates. One can expect that Cho interacts with hydrophobic amino acids of proteins developing protein/Cho complexes that template structurally different protein aggregates compared to those formed under lipid-free conditions.

Our results, as well as recently reported by our group experimental findings, demonstrate that such amyloid aggregates can be endocytosed by cells. This causes substantial damage of the cell endosome and leakage of the aggregates in the cytosol where these protein specimens damage the endoplasmic reticulum and mitochondria. These conclusions could be made by the observed increase in the expression of Chmp1, Gal3, and TFEB factors in the neuronal cells exposed to both α-Syn and α-Syn/Cho fibrils. Although the molecular mechanism of this process is unclear, our findings also demonstrate that both macrophages and NK cells could degrade such aggregates. Our results showed that macrophages and NK cells respond differently to oligomers and fibrils. Specifically, NB cells preferably endocytosed fibrils compared to monomers and oligomers, whereas macrophages, on the opposite, demonstrated the strongest intake of monomers and oligomers compared to fibrils. These results suggest that both macrophages and NK cells could be considered as a new therapeutic approach for the efficient clearance of amyloid aggregates.

Recently, Sheng group showed that meningeal lymphatic vessels (MLV) could be used to deliver nanocomplexes that were capable of inhibiting α-syn aggregation to the brain.^79^ Specifically, the natural killer cell membrane biomimetic nanocomplex (BLIPO–CUR) allowed for targeting damaged neurons suppressing levels of reactive oxygen species simultaneously inhibiting the aggregation of α-syn and, consequently, decelerating the progression of PD. These results showed that modification of lipid membranes of NK cell and macrophages could be used to enable their transition through the blood–brain barrier. These findings also indicate that utilization of several rather than one therapeutic strategy is likely to be the most effective approach to treat PD.

## Conclusions

Our results showed that PS and Cho drastically accelerated the aggregation rate of α-Syn, which resulted in the appearance of significantly more toxic oligomers and fibrils than those formed in the lipid-free environment. These aggregates, as well as α-Syn monomers, can be endocytosed by macrophages and NK cells. We also found that both macrophages and NK cells could degrade protein monomers and aggregates. Furthermore, macrophages could internalize α-Syn monomers, oligomers, and fibrils more efficiently than NK cells. The exposure of both macrophages and NK cells to monomeric α-Syn and its aggregates induced cell proliferation and drastically changed the cytokine and chemokine profiles of these cells. These results suggest that both macrophages and NK cells could help to decelerate the spread of neurodegeneration and, consequently, slow the progression of neurodegenerative diseases.

## Supporting Information Available

ThT kinetics of protein aggregation (Figure S1) (PDF)
