# Supplementary material for: Macrophages and Natural Killers Degrade α-Synuclein Aggregates
Source: Mol Pharm. 2024 Apr 18;21(5):2565–76. doi: 10.1021/acs.molpharmaceut.4c00160 (PMC11080468; doi:10.1021/acs.molpharmaceut.4c00160)
Supplement: Supplementary file 1 — mp4c00160_si_001.pdf [file mp4c00160_si_001.pdf]

Macrophages and Natural Killers Degrade α-Synuclein Aggregates

Mikhail Matveyenka[1], Kiryl Zhaliazka[1] and Dmitry Kurouski*[1,2]

1. Department of Biochemistry and Biophysics, Texas A&M University, College Station, Texas 77843, United States

2. Department of Biomedical Engineering, Texas A&M University, College Station, Texas, 77843, United States

Corresponding author: Dmitry Kurouski; email: dkurouski@tamu.edu; phone: 979-458-3448.

Supporting Information

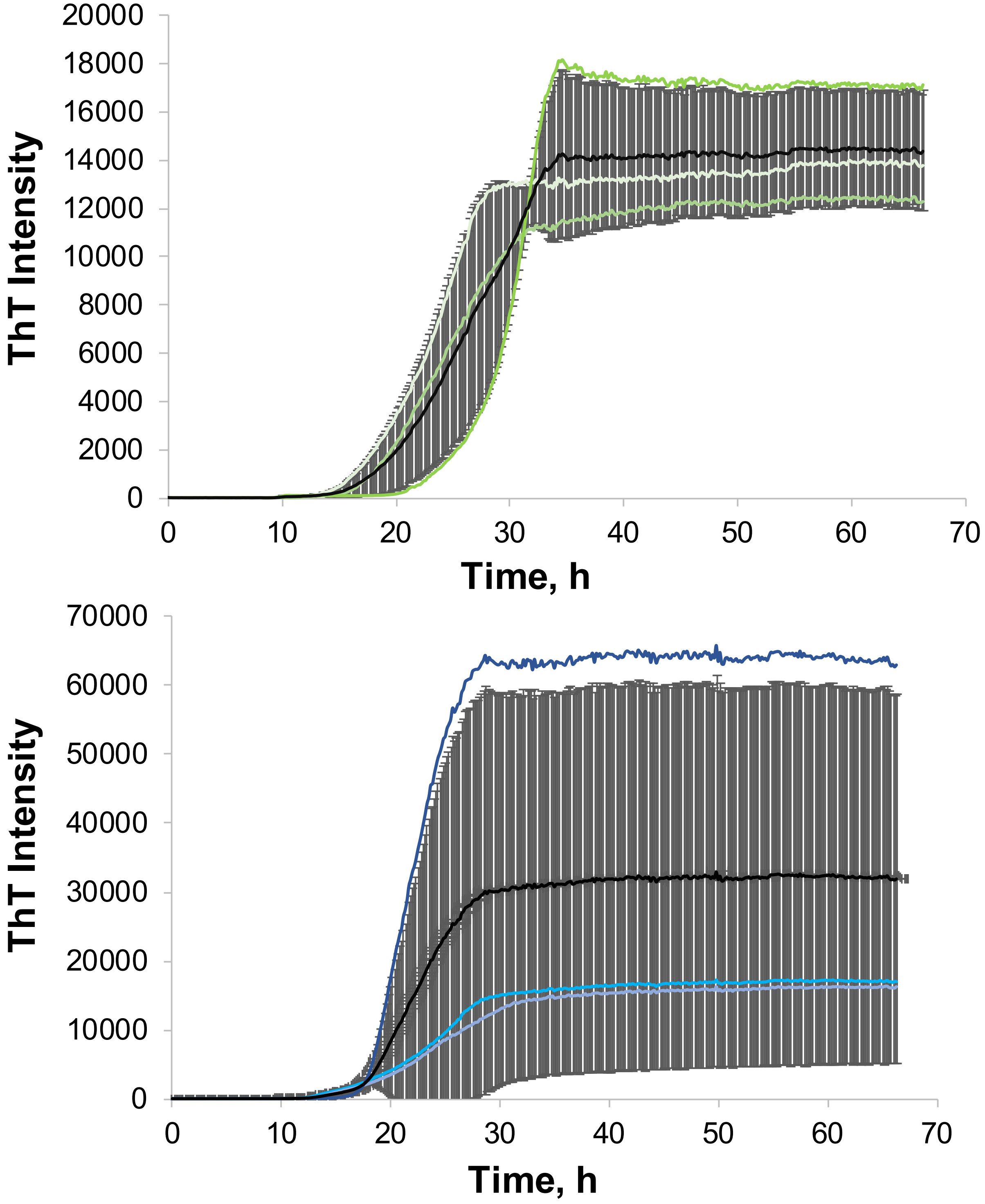

Figure S1. Individual ThT kinetics (colored lines) and averaged ThT (black) of α-Syn aggregation in the lipid-free environment (top) and in the presence of PC:Cho mixture.
